# Rapid clinical identification of vulnerable older adults in acute and emergency care: narrative review of short geriatric screening tools

**DOI:** 10.3389/fmed.2026.1815638

**Published:** 2026-05-21

**Authors:** Danilo Češljarac, Erik Lipej, Brina Skaza, Ana Spirkoska Mangaroska, Mišo Šabovič, Barbara Eržen

**Affiliations:** 1 Division of Internal Medicine, Faculty of Medicine, University of Ljubljana, Ljubljana, Slovenia; 2 Department of Vascular Diseases, University Medical Centre Ljubljana, Ljubljana, Slovenia

**Keywords:** acute care, clinical intervention, emergency department, geriatric screening, multi-domain screening instruments, older patients, short questionnaires, two-step approach

## Abstract

**Background:**

Older adults presenting to emergency departments or acute wards are at increased risk of functional decline, delirium, falls, prolonged hospital stay, and early mortality. Early recognition of vulnerability is crucial for timely preventive interventions, yet specific geriatric assessment is often not performed at admission due to time limitations.

**Aim:**

To provide a clinically focused synthesis of short geriatric screening tools and present a practical two-step model linking early screening with targeted clinical action.

**Methods:**

This narrative review summarizes brief screening instruments that can be completed within minutes and are suitable for routine use. A targeted literature review identified tools used and evaluated available evidence on predictive accuracy, feasibility, and clinical integration.

**Results:**

Rapid tools such as ISAR, TRST, PRISMA-7, and APOP allow early identification of patients at risk of functional decline, readmission, or short-term mortality. Multidomain instruments, including HARP, SHERPA, and ISAR-HP, provide broader prognostic information, supporting discharge planning and targeted follow-up. Predictive accuracy is generally moderate, with the area under the curve (AUC) typically between 0.60 and 0.70. Clinical impact depends on embedding screening into routine workflows and linking results to actionable interventions such as early mobilization, cognitive assessment, medication review, or referral to geriatric specialists. Clinical integration seems to require a tiered, two-step approach: rapid screening upon admission, followed by a comprehensive multidomain assessment on the ward.

**Conclusion:**

Short geriatric screening tools are essential in acute care. A two-step approach appears to be a practical and clinically reasonable strategy for optimizing early detection and linking screening to targeted interventions. However, further research is needed to validate its effectiveness and define the optimal implementation strategy.

## Introduction

Europe’s population is aging, with more than 20% currently aged 65 years or older. This proportion will continue to increase in the coming decades (1, 2). The aging of the European population has a significant impact on already overburdened healthcare systems, as evident by the increasing number of hospitalizations and emergency department visits among older patients (3). Therefore, hospitals are under increasing pressure to provide safe and comprehensive care for older patients.

For older patients, hospitalization is an important stress factor (4). During hospital care, new problems often occur, or existing conditions worsen. Examples of such problems include delirium, falls, incontinence, functional and cognitive decline, as well as other complications not necessarily directly related to the reason for admission (5, 6). Hospital-associated functional decline is very common and can threaten the long-term independence of older patients (6, 7). Moreover, longer hospital stays, higher risk of institutionalization, and higher mortality are also common (7–9). Older patients are often frail, meaning they have reduced physiological and functional reserve. This is reflected in a reduced ability to respond to stressors, increasing the risk of complications during hospitalization (10, 11). In the emergency setting, approximately 20%–40% of older patients are considered frail, with even higher proportions reported in some meta-analyses (12–14). Therefore, early identification of vulnerability is essential, as it enables the timely introduction of targeted preventive measures, leading to a reduced risk of complications and irreversible functional decline.

Emergency departments and hospital wards are usually not adapted to the complex needs of older patients. The clinical presentation of acutely ill older patients often includes a combination of medical, cognitive, functional, and social problems. On the other hand, work processes are mainly focused on rapid assessment and high patient throughput (15). Comprehensive geriatric assessment (CGA) is the gold standard for identifying at-risk older patients, but its routine use in acute settings is often limited due to time and staffing constraints (16). Therefore, short screening tools are used in practice to enable rapid identification of vulnerability at arrival to the emergency department or shortly after hospital admission. These tools are based on a few key indicators, such as functional decline, cognitive problems, mobility, previous hospitalizations, and polypharmacy. Furthermore, they require little time to perform and minimal training. Short screening tools can also be used by healthcare workers without specialized geriatric knowledge. Their role is screening, and they are not a substitute for CGA. Short screening tools are used to identify high-risk older patients who, in the second step, may benefit from either CGA or specialized geriatric care. European guidelines recommend routine screening (17, 18), but its implementation in daily clinical practice remains insufficient. In addition to the impact on the individual patient, early identification of vulnerability also has broader system-level implications, as it can contribute to better organization of care for older patients and more rational use of healthcare staff.

Therefore, in this narrative review, we focus on short geriatric screening tools and propose a simple two-step approach that may help guide clinical decision-making: screening with short tools and the subsequent involvement of CGA or targeted geriatric care.

## Methods

This article presents a pragmatic narrative review of brief geriatric screening questionnaires, commonly used in emergency departments and during early acute hospital admission. For the purpose of this review, emergency department and early acute inpatient settings are considered together, as many screening tools apply across both contexts. The aim is to provide a clinically useful overview of tools that can realistically be applied in routine practice, rather than providing an exhaustive summary of all available studies.

Therefore, the concentration was placed on screening instruments regularly used by frontline staff that support everyday clinical decision-making in busy, time-pressured acute care settings. A narrative approach was considered the most suitable, as the existing literature is highly heterogeneous, having substantial differences in study design, clinical setting, outcomes, and the composition of screening tools themselves. In this context, our aim is not to perform a formal statistical comparison, but to describe how these tools perform and how feasible they are when applied in real-world clinical practice. As this is a narrative review, a formal risk of bias assessment was not performed. However, key methodological aspects of included studies were considered, including study design, sample size, outcome definitions, and consistency of findings.

### Search strategy

A targeted and purposive search of the PubMed, Scopus and Embase databases was performed for studies published up to and including March 2026, using terms related to older adults, hospitalization, emergency care, screening tools, frailty, functional decline, delirium, falls, readmission, and mortality. PubMed, Scopus and Embase were selected as the primary databases as they index most of the peer-reviewed literature relevant to geriatric and emergency medicine. Search terms included combinations of: “geriatric screening,” “short screening tools,” “frailty,” “emergency department,” “acute hospital care,” “functional decline,” “delirium,” “falls,” “readmission,” and “mortality.” Moreover, they also included the additional related terms: “screening instrument,” “risk stratification,” “risk prediction,” “clinical screening,” “vulnerability,” “hospital admission,” “older adults,” “functional impairment,” “institutionalization,” “adverse outcomes,” etc. Outcome-related terms (e.g., functional decline, delirium, falls, readmission, mortality) were selected because they represent clinically relevant adverse outcomes in older patients in emergency and acute care settings. The terms were not used simultaneously in a single search query, but rather combined in different ways to get as many useful articles as possible. An example of a search string used was: (“geriatric screening” OR “frailty screening” OR “short screening tools”) AND (“emergency department” OR “acute hospital care”) AND (“older adults” OR “elderly”). To ensure that key articles were not missed, additional relevant articles were identified through targeted Google Scholar searches, as well as by reviewing the reference lists of key publications, systematic reviews, and European and international guidelines (12, 15, 19). Titles and abstracts were screened with a pragmatic focus on short screening tools that can be completed within a few minutes, intended for use at emergency department presentation or early during acute hospital admission. All included articles were published in English. The search strategy was deliberately focused.

### Eligibility criteria

The research concentration was placed on short geriatric screening tools used in emergency departments or during early acute hospital care. Titles and abstracts were screened first, and full-text articles were reviewed only when relevant. Studies of patients aged 65 years or older that evaluated brief geriatric screening questionnaires were included. Judgment-based frailty tools, such as the Clinical Frailty Scale and the FRAIL scale, are discussed conceptually but were not included in the tables. Clinically relevant outcomes were comprised of functional decline, delirium, falls, length of hospital stay, institutionalization, readmission, and short-term mortality (12, 15, 19–21). Moreover, validation studies of screening tools used at hospital admission were also included (e.g., ISAR-HP), when the reported outcomes were comparable (20, 21). Some screening tools were first developed and tested in community-dwelling older adults and are now also used in acute and emergency hospital care; therefore, the original community-based studies were also included (22, 23). The following criteria were used for exclusion: studies limited to specific disease groups, studies conducted only in long-term care or rehabilitation settings, studies without reported clinical outcomes, and articles that were not available in full text.

### Data synthesis

As this is a narrative review, findings were summarized descriptively, emphasizing clinical usefulness and feasibility rather than quantitative comparison. The focus was on the tools’ practicality and usefulness in everyday clinical practice, rather than on formal quality ratings or statistical comparisons between tools (13). However, interpretation of the evidence also considered key methodological limitations of the included studies, including heterogeneity in study design, patient populations, outcome definitions, sample size, and extent of external validation.

As this is a pragmatic narrative review, formal quantitative tracking and counting of relevant articles at each step were not performed (e.g., the exact number of articles identified, reviewed, and excluded). Nevertheless, study selection and synthesis were guided by clinical relevance and consistency of findings across studies. The final narrative synthesis was based on 52 references considered most relevant to the scope and clinical focus of this review.

## Overview of screening tools

Identifying frailty early in older adults presenting to emergency departments or acute wards is critical to prevent functional decline, delirium, falls, and early mortality. Clinicians frequently use judgment-based instruments such as the Clinical Frailty Scale (CFS) and the FRAIL scale. While these tools are widely applied and supported by European guidelines, they rely heavily on clinical experience, which can introduce variability, particularly in busy acute care settings or when used by staff without geriatric training. For this reason, our review focuses on standardized, questionnaire-based tools that non-specialist staff can apply reliably.

Short geriatric screening questionnaires help clinicians quickly spot older patients at higher risk of adverse outcomes during their stay in the emergency department or hospital. They are brief, structured, and easy for non-specialist staff to use in routine practice.

The first group includes rapid triage tools, typically with only a few items that can be completed in 1–2 min. Their main goal is to provide early warning of vulnerability so that high-risk patients can be identified promptly. Common examples are the Identification of Seniors at Risk (ISAR) and the Triage Risk Screening Tool (TRST). ISAR has six yes/no questions covering recent functional decline, memory issues, polypharmacy, and recent hospitalization, and can typically be completed in about a minute (24). TRST focuses on cognition, mobility, living situation, and previous emergency visits, helping to flag patients at risk of early return visits, readmission, or complex discharge needs (25). PRISMA-7, although originally developed for community-dwelling older adults, is now increasingly used at emergency department admission as a simple frailty screen (23). However, most validation data originate from community-based populations, whereas evidence from emergency department settings remains more limited. It provides a quick sense of mobility limitations, functional dependence, and the need for assistance with daily activities. The Acutely Presenting Older Patient (APOP) screener was created specifically for emergency hospital settings. It includes age, illness severity, cognition, and mobility. APOP is therefore used to identify patients at high risk of functional decline or death within 90 days and can be completed in just a few minutes (26). Although these tools differ in content and design, they all aim to highlight patients at higher risk who may benefit from closer monitoring or further assessment. Rapid tools emphasize speed and sensitivity, so specificity is usually lower–this is reflected in the commonly used cut-off values: ISAR ≥ 2, TRST ≥ 2, and PRISMA-7 ≥ 3.

The second group of tools consists of multidomain screening instruments. These take slightly longer to complete but cover a broader range of clinical domains. The Hospital Admission Risk Profile (HARP), for example, combines age, a brief cognitive test, and basic activities of daily living to estimate the risk of functional decline during hospital stay and anticipate complex discharge needs (27). ISAR-HP, developed specifically for hospitalized older adults, is a short four-item tool, focusing on premorbid mobility and functional status, with good predictive accuracy for functional decline during admission (20). Some tools are designed to predict specific outcomes. For instance, the Variable Indicative of Placement (VIP) tool aims to identify patients at higher risk of institutionalization, including items related to mobility, cognition, continence, and social circumstances (28, 29). The SHERPA tool provides a broader, multidimensional profile that covers mobility, medication use, falls, cognitive concerns, and activities of daily living. It was developed to predict functional decline after discharge and to support early discharge planning (30).

More recently, digital instruments such as the interRAI ED Screener have shown promising results, although current evidence is still emerging and largely based on recent single-country validation cohorts (31).

In addition to bedside tools, administrative instruments such as the Silver Code rely on routinely collected demographic and clinical data and require no direct patient contact. These tools can support screening in settings with advanced digital infrastructure, though they are less suitable for real-time assessment upon arrival in the emergency department. Other instruments have also been described in the literature, including SEGA, BRIGHT, TaGA, ICEBERG, and FIND-NEEDS. These tools are not all designed for the same purpose. Some, such as SEGA and BRIGHT, are older and are now used less often in emergency departments, partly because they have more limited validation and are not widely used in current practice. Others take a broader look at the patient and cover several geriatric domains, which usually require more time and clinical input, so they do not fully fit the concept of short screening tools. This is the case for tools such as TaGA and ICEBERG, which are therefore less suitable for rapid screening in busy acute care settings. FIND-NEEDS, on the other hand, focuses more on identifying specific geriatric problems, such as functional, cognitive, or nutritional issues, and is closer to tools used before a full geriatric assessment than to those used to estimate short-term risk in acute care (32–36).

Table 1 summarizes the main characteristics of short geriatric screening tools, including the number of items, time required, domains assessed, recommended setting, cut-off values, and intended outcomes.

**TABLE 1:** Tool characteristics, domains covered, and suggested clinical use.

Tool	Items/time	Score range and cut-off	Administration	Domains covered	Typical clinical use
ISAR – Identification of Seniors at Risk (24)	6 items/1–2 min	0–6; cut-off ≥ 2	Patient interview	Functional status, cognition, sensory impairment, polypharmacy, recent hospitalization	Rapid triage; identification of older adults at risk of adverse outcomes in the ED
TRST – Triage Risk Screening Tool (26)	5 items/1–2 min	High risk ≥ 2	Nurse-administered	Cognition, mobility, living situation, previous ED use, medications	Rapid triage; identification of patients at risk of ED return visits and early readmission
PRISMA-7 (23)	7 items/<1 min	Frailty ≥ 3	Patient interview	Age, sex, mobility, need for assistance, health status	Rapid triage; frailty screening at ED arrival
APOP screener – Acutely Presenting Older Patient (26)	7 variables/1–2 min	Algorithm-based high-risk classification	Nurse or physician	Age, functional status, cognition, recent healthcare use, and illness acuity	Prediction of 90-days mortality and functional decline in ED patients
HARP – Hospital Admission Risk Profile (27)	3 components/ 3 min	Low/intermediate/ high risk	ADL interview + brief cognitive test	Age, cognition, ADL function	Prediction of in-hospital functional decline; support for discharge planning
ISAR-HP (20)	4 items/3 min	High risk ≥ 2	Nurse-administered	Premorbid mobility, functional status, ADL	Prediction of functional decline during hospitalization and short-term mortality
VIP – Variable Indicative of Placement (28, 29)	7 items/3–4 min	0–7; no universal cut-off – higher scores indicate higher placement risk	Nurse-administered	Mobility, cognition, continence, social situation	Identification of patients at risk of institutionalization
SHERPA (30)	7 items/5 min	0–14; higher = higher risk	Nurse or geriatric team	Mobility, falls, medications, cognition, ADL function	Prediction of functional decline; support for discharge planning
interRAI ED Screener (31)	7 items/2–3 min	Automated risk level	Digital/EHR-supported	Function, cognition, mobility, clinical stability, and social context	Use in digitally supported settings; early identification of patients at risk of prolonged stay, functional decline, or unplanned readmission

ISAR, Identification of Seniors at Risk; TRST, Triage Risk Screening Tool; APOP, Acutely Presenting Older Patient; PRISMA-7, Program of Research to Integrate Services for the Maintenance of Autonomy; HARP, Hospital Admission Risk Profile; SHERPA, Score Hospitalier d’Évaluation du Risque de Perte d’Autonomie; VIP, Variable Indicative of Placement; ISAR-HP, Identification of Seniors at Risk–Hospitalized Patients; interRAI ED Screener, International Resident Assessment Instrument Emergency Department Screener; ED, emergency department; ADL, activities of daily living; IADL, instrumental activities of daily living; CGA, comprehensive geriatric assessment; LOS, length of stay; MMSE, Mini-Mental State Examination; AUC, area under the curve.

## Predictive performance

Short geriatric screening tools vary in their ability to predict outcomes such as functional decline, prolonged hospital stay, readmission, and short-term mortality. Systematic reviews generally report moderate prognostic performance, with typical area under the curve (AUC) values around 0.60–0.70 for composite adverse outcomes (12, 37). Differences within this range are relatively small and may have limited clinical significance. These tools are therefore more useful for risk stratification than for precise prediction. As shown in Table 2, performance varies partly because studies use different cut-off values, reflecting a balance between sensitivity and specificity, depending on the intended clinical use.

**TABLE 2:** Validation and predictive performance of short geriatric screening tools.

Tool	Key validation studies	Setting/ population	Sample size	Predicted outcomes	Predictive performance (reported metrics)
ISAR	McCusker et al.; Salvi et al.; Asomaning and Loftus (19, 24, 33)	ED patients ≥ 65 years (Canada, Europe, USA)	*n* ≈ 200–1,500+	Functional decline, ED return, readmission, mortality	Sensitivity ∼0.75–0.90; specificity ∼0.35–0.60; AUC typically ∼0.58–0.62 (overall ∼0.60)
ISAR-HP	Hoogerduijn et al. (20)	Hospitalized patients ≥ 65 years	*n* ≈ 400	Functional decline, 90-days mortality	AUC ∼0.68–0.71 for functional decline; moderate predictive validity
TRST	Meldon et al.; Carpenter et al. (12, 25)	Older ED patients, USA	*n* = 1,663	Readmission, ED revisit, institutionalization	High sensitivity with lower specificity; AUC ∼0.60–0.65
APOP	de Gelder et al.; Blomaard et al. (26, 36)	ED patients ≥ 70 years (Netherlands)	*n* = 2,601; *n* ≈ 640	90-days functional decline and mortality	AUC ∼0.70–0.78 in pre-COVID cohorts; lower during COVID
HARP	Sager et al.; subsequent validation studies (27)	Acute admissions ≥ 65 years	*n* = 139 (original); larger datasets	In-hospital functional decline, discharge needs	Reported AUC mostly ∼0.63–0.70; moderate predictive validity
SHERPA	Cornette et al. (30)	Acute geriatric admissions, Switzerland	*n* = 505	Functional decline after discharge	AUC ∼0.70–0.73; sensitivity ∼0.68–0.90; specificity ∼0.54–0.71
interRAI ED Screener	Mowbray et al. (31)	ED patients ≥ 65 years, Canada	*n* = 5,629	Need for CGA, prolonged ED stay, and discharge outcomes	Sensitivity 0.93; AUC ∼0.70 for composite adverse outcomes
PRISMA-7	Raîche et al. (23)	Community-dwelling adults ≥ 65 years	*n* > 1,000	Frailty detection	AUC ∼0.74–0.80 for frailty in community settings; evidence in ED populations remains limited
VIP	Vandewoude et al.; van Loon et al. (28, 29)	Older adults ≥ 65 years in ED or at hospital admission (Belgium, Netherlands)	*n* = 3,920; *n* ≈ 450	Placement risk, length of stay, discharge destination	AUC ∼0.65–0.72 where available; moderate prediction of institutionalization

ED, emergency department; EHR, electronic health record; GEDI, Geriatric Emergency Department Intervention; GEDA, Geriatric Emergency Department Accreditation; CFIR, Consolidated Framework for Implementation Research; CGA, comprehensive geriatric assessment; LOS, length of stay; ADL, activities of daily living. Reported performance metrics represent approximate ranges derived from key validation studies. Different studies reported different metrics (e.g., AUC, sensitivity, specificity, PPV); original metrics are presented to avoid loss of information.

Differences in performance across studies are also likely related to variation in patient populations and clinical settings. Patients differ in age, level of frailty, and comorbidity, which can influence outcomes. Clinical environments (emergency department vs. inpatient wards) and definitions of outcomes also vary between studies. As a result, findings are often inconsistent and should be interpreted with caution. Some tools prioritize sensitivity, while others favor specificity, which further contributes to differences in reported performance. In addition, much of the available evidence is based on observational, often single-center studies, which may further limit comparability and generalizability. In addition, differences in reported performance may reflect how tools are designed and what they capture. Some focus mainly on functional status, while others include cognitive or clinical factors, which can influence which patients are identified as high risk. Results may also vary because studies use different cut-off values and outcome definitions, making direct comparison more difficult.

Rapid triage tools are designed for quick risk detection and therefore tend to prioritize sensitivity over specificity. ISAR, for instance, often shows high sensitivity (0.75–0.90) but lower specificity, with overall AUC values around 0.60–0.65 (11, 24, 38–40). TRST follows a similar pattern, with high sensitivity and AUC values mostly in the 0.60–0.65 range, particularly for repeat emergency visits and early readmission (12, 25). The APOP screener generally performs better than other brief tools. In pre-COVID cohorts, AUC values ranged from 0.70 to 0.78 for 90-days functional decline and mortality, though slightly lower performance has been reported in some COVID-period studies (26, 41).

Multidomain tools that assess functional, cognitive, and mobility domains tend to show more balanced performance. HARP and ISAR-HP typically reach AUC values in the mid-0.60s to low-0.70s for predicting functional decline and discharge outcomes (20, 27). Similar results have been reported for the Variable Indicative of Placement (VIP) tool in predicting institutionalization and complex discharge (28, 29). SHERPA also performs well, with AUC values around 0.70–0.73 and reasonable sensitivity and specificity (30, 42). More recently, digital instruments, such as the interRAI ED Screener, have shown promising results, with high sensitivity and good predictive accuracy for longer emergency department stays, need for comprehensive geriatric assessment (CGA), and discharge destination (31).

Most validation studies focus on sensitivity, specificity, and AUC, while other measures, such as calibration or decision analysis, are rarely reported. In addition, different studies report different performance metrics (AUC, sensitivity, specificity), which further limits direct comparison between tools. Because of this, direct comparison between tools is difficult, and clear conclusions about their relative performance are limited. It also limits the ability to fully assess the clinical utility of screening tools or to determine optimal cut-off values for different settings. Future studies should report these measures more consistently to support informed implementation. In practice, differences between tools are usually small. Most show similar ranges of predictive accuracy, which means that outcomes are probably influenced less by minor differences in AUC and more by how screening is actually used in everyday clinical workflows. Importantly, these differences may partly reflect variation in study design and populations rather than true differences in tool performance.

It is important to distinguish between risk prediction and actual clinical benefit. Many screening tools identify older patients at increased risk of adverse outcomes during hospitalization, but screening alone does not improve clinical outcomes unless it is followed by timely and targeted preventive interventions. Rapid tools are best suited for early risk identification, whereas multidomain instruments provide broader prognostic information and may help guide functional decline management and discharge planning.

## Feasibility in practice

The feasibility of short geriatric screening tools largely depends on how well they fit into routine emergency and acute care workflows. Tools are more likely to be used consistently when they are brief, easy to score, and can be completed by nurses or other frontline staff without specialized training (13, 18). Instruments such as ISAR, TRST, PRISMA-7, and APOP are well-suited for use during emergency department triage, as they provide rapid assessments without significantly disrupting patient flow.

Screening uptake improves when tools are integrated into existing documentation and recorded directly in electronic health records. Digital integration not only increases the visibility of screening results but also facilitates communication across the clinical team (40–43). In contrast, more complex tools, such as SHERPA or the interRAI ED Screener, may be harder to implement in very busy emergency departments or in settings with limited digital infrastructure.

Implementation studies show that feasibility is influenced not only by the instrument itself but also by organizational factors. Hospitals that have established structured geriatric care pathways, dedicated geriatric emergency staff, or models like the Geriatric Emergency Department Intervention (GEDI) report higher screening uptake and better recognition of frailty-related risks (44, 45). Clear assignment of responsibilities, staff training, and defined follow-up processes are essential to support sustained use in routine practice.

## Tiered (two-step) approach

In this review, we present the two-step model as a practical way to organize existing screening tools within routine acute care workflows.

A tiered, two-step approach can be understood as a practical way to organize geriatric screening tools within routine emergency and acute care workflows. Since no single instrument provides both high sensitivity and high specificity, some guidelines and expert recommendations suggest a stepwise process: a brief initial screen is performed first, followed by a more detailed assessment only when indicated (17, 18). Very short tools such as ISAR, TRST, PRISMA-7, and APOP can be completed in 1–2 min and are well-suited for first patient contact. Their main purpose is to flag vulnerability related to functional decline, cognitive impairment, reduced mobility, or recent hospitalization.

A positive result on the initial screen helps prioritize patients who may benefit from further assessment. Subsequent evaluations can be tailored to local resources and may include a targeted mobility assessment, delirium screening (e.g., 4AT), brief cognitive testing, medication review, or focused nursing and social evaluations. This approach allows early identification of key risks while avoiding unnecessary assessments for patients at lower risk.

More detailed multidomain tools, such as HARP, ISAR-HP, SHERPA, or VIP, can be applied selectively to predict functional decline, discharge needs, or risk of institutionalization. In this context, comprehensive geriatric assessment is reserved for patients with clear vulnerability or complex needs. Digital solutions can further support this model by enabling automated risk stratification and timely referral for geriatric input (46–48).

Based on the available evidence and practical experience in acute care settings, the two-step (tiered) approach can be considered a practical and clinically reasonable strategy for geriatric screening can be considered. This model may allow rapid identification of patients at risk using brief initial tools, while reserving more detailed multidomain assessments for those with demonstrated vulnerability. Therefore, this approach may be useful in routine practice, as it balances efficiency, early detection, and targeted intervention, ensuring that limited resources are directed where they are most needed. Overall, the two-step process may help hospitals to turn early detection into actionable care. Moreover, it can help improve patient outcomes while maintaining efficiency in fast-paced clinical settings.

In practice, the successful implementation of this model depends on how well screening is integrated into everyday workflows and linked to clear clinical actions. Therefore, the following section focuses on practical barriers and solutions for routine implementation.

## Implementation in routine practice: barriers and solutions

Although guidelines recommend routine screening of older adults in emergency and acute care settings (17, 18), implementation in daily practice remains uneven. Emergency departments are often crowded and understaffed, with staff working under time pressure. In such conditions, screening is easily skipped, especially when it is not part of standard triage or admission routines (43, 44). Responsibility is also not always clear. Nurses and physicians may assume that screening is someone else’s responsibility, which reduces consistency.

Organizational factors play an important role. In many hospitals, documentation is still partly paper-based or not fully integrated into electronic health records. This makes screening harder to perform and results less visible to the team. Limited digital support also increases administrative workload. Differences in staffing and access to geriatric expertise further contribute to variability between hospitals and health systems (43, 44). These problems are more pronounced in smaller or resource-limited settings.

Studies show that implementation improves when screening is built directly into routine workflows (44, 49). Tools are used more consistently when they are included in triage or admission forms. Digital systems can help by providing automatic scoring and clear visibility of results for the whole team (43, 44). In these settings, screening becomes part of routine care rather than an additional task.

Teamwork is equally important. Models, such as the Geriatric Emergency Department Intervention (GEDI), show that collaboration between nurses, geriatricians, physiotherapists, pharmacists, and other professionals supports earlier recognition of risk and more appropriate follow-up (45, 50). Strong organizational support and clear leadership help sustain these processes over time (51, 52). Without defined pathways, screening results often do not lead to action.

Short, targeted training has been shown to increase adherence to screening pathways. Short training sessions and practical workshops can improve confidence in recognizing frailty and interpreting screening results (18). This is especially useful in departments without dedicated geriatric teams. Staff need to know not only how to screen, but also what to do next. Screening should therefore be linked to clear interventions such as early mobilization, delirium prevention, medication review, or referral for comprehensive geriatric assessment.

Screening alone is unlikely to improve clinical outcomes, unless it is linked to clear clinical actions. This also suggests that proposed models, such as the two-step approach, should be interpreted as organizational strategies rather than interventions with proven effects on clinical outcomes. Studies suggest that the benefit of screening depends on whether it leads to targeted interventions that encompass comprehensive geriatric assessment, early mobilization, medication review, or multidisciplinary care. This highlights that screening should be integrated into structured care pathways rather than used as a standalone tool. In practice, effective implementation depends on clear responsibility, defined follow-up actions, and organizational support.

A simple example of how this two-step approach may work in practice is shown below:

An older patient presenting to the emergency department undergoes a brief screening (e.g., ISAR). A positive result (e.g., ISAR ≥ 2 or another tool-specific threshold) indicates an increased risk of adverse outcomes. In this case, a second step is initiated, including targeted assessments such as mobility evaluation, delirium screening, medication review, and, when appropriate, a comprehensive geriatric assessment. Based on these findings, appropriate interventions are introduced, such as early mobilization, delirium prevention, or discharge planning. This approach enables a direct link between screening, further assessment, and concrete clinical actions.

In practice, tools can be selected according to clinical purpose (12, 13, 17, 20, 21, 23–38). The typical clinical use of individual screening tools is summarized in Table 1 and reflects their role in different clinical contexts, including rapid triage, risk prediction, discharge planning, and identification of patients at risk of institutionalization.

Matching the tool to the clinical goal helps keep the process simple and efficient. Brief tools can be used for early identification, while multidomain tools can be reserved for patients who need a more detailed assessment. Overall, successful implementation depends more on the workflow than on selecting a single tool. Implementation studies suggest that screening works best when a tiered approach is integrated into routine admission processes, supported by digital systems, and linked to clear clinical actions.

## Future directions

With population aging across Europe and an increasing number of older patients presenting to emergency and acute care, future practice will need to focus on translating screening results into timely, actionable interventions. The goal is to ensure that routine, computer-supported screening leads to appropriate, individually tailored clinical responses. Screening is likely to become increasingly embedded within structured care pathways, such as acute frailty units, geriatric emergency teams, and senior-friendly hospital models (17, 49).

Age-friendly hospital initiatives are also expected to play a larger role by adapting care processes, the physical environment, and staffing to better meet the needs of older patients. Greater attention will be given to the patient’s own perspective, including baseline function, recent changes, and personal goals. The implementation of the patient’s perspective may help interpret screening results and guide follow-up decisions (10, 20).

Further research is needed to better understand the impact of geriatric screening on long-term outcomes, including functional status after discharge, risk of institutionalization, and medication-related harm. Additional studies should evaluate its cost-effectiveness in relation to complications, length of stay, and readmissions. Future research should also address specific practical questions, such to what extent general interventions (e.g., early mobilization, delirium prevention, medication review) introduced after a positive screening result reduce in-hospital complications; and whether a two-step approach combining screening with comprehensive geriatric assessment and individualized interventions provides additional benefit compared to a uniform approach. It also remains unclear how screening can be most effectively integrated into routine clinical workflows to ensure consistency between results and concrete clinical actions. Even as digitalization and automated decision support become more widespread, clinical judgment and person-centered care will remain essential to ensure meaningful, patient-focused outcomes.

Recent studies published after the initial search period largely confirm previous findings, without identifying a clearly superior screening tool or substantially changing the current evidence base.

## Limitations

This narrative review has several limitations. First, it is not a systematic review: databases and key reference lists were screened, but relevant studies in other databases or languages may have been missed. Clinical relevance and clarity were prioritized over exhaustive coverage. Second, the included studies varied in design, populations, outcomes, and definitions of frailty or functional decline. Such heterogeneity limited direct comparisons and prevented quantitative synthesis. Many validation studies were small, single-centered, or focused on specific populations. In addition, most included studies were observational and therefore subject to potential selection bias. The generalizability of findings may be limited, as tool performance can vary across different patient populations (e.g., frailty level, comorbidity) and healthcare systems with different resources and organization of care. Moreover, differences in reported performance may partly reflect these methodological and population differences rather than true differences between screening tools. Third, predictive performance was reported inconsistently, with varying follow-up periods (e.g., 30 days, 90 days, or during hospital stay) and different metrics (sensitivity, specificity, AUC), making cross-tool comparisons difficult. Evidence on implementation is also uneven: some tools are well-studied in emergency departments, while others were tested mainly in inpatient or community settings. Results from countries with well-established geriatric services may not apply to settings with fewer specialists or limited resources. Furthermore, external validation of several tools remains limited, which should be taken into account when interpreting their performance. Finally, this review reflects evidence available up to 2026; therefore, newer digital or AI-supported approaches may not yet be captured. Despite these limitations, it provides a practical, clinically grounded overview of short geriatric screening tools for emergency and acute care.

## Conclusion

Early identification of vulnerability in older patients in emergency and acute care is strongly supported by current literature. Rapid triage tools allow clinicians to quickly identify high-risk patients. Multidomain instruments, on the other hand, provide additional information relevant to functional decline and discharge planning. In settings with limited geriatric resources, a tiered approach may direct specialist input where it is most needed. However, screening alone is not sufficient to improve outcomes and must be followed by timely, targeted clinical interventions. Early recognition when linked to appropriate interventions, may reduce complications, length of stay, and the need for institutional care. As geriatric care models and digital systems continue to evolve, short screening tools will likely remain an important part of routine practice. However, further research is needed to clarify long-term outcomes, cost-effectiveness, and the best ways to translate screening results into concrete clinical actions within everyday workflows.
