# Correction: Rapid clinical identification of vulnerable older adults in acute and emergency care: narrative review of short geriatric screening tools

**DOI:** 10.3389/fmed.2026.1898845

**Published:** 2026-06-29

**Authors:** Danilo Češljarac, Erik Lipej, Brina Skaza, Ana Spirkoska Mangaroska, Mišo Šabovic, Barbara ErŽen

**Affiliations:** 1 Division of Internal Medicine, Faculty of Medicine, University of Ljubljana, Ljubljana, Slovenia; 2 Department of Vascular Diseases, University Medical Centre Ljubljana, Ljubljana, Slovenia

**Keywords:** acute care, clinical intervention, emergency department, geriatric screening, multi-domain screening instruments, older patients, short questionnaires, two-step approach

In the published article, the **Funding** statement was erroneously published as:

The author(s) declared that financial support was not received for this work and/or its publication.

The correct **Funding** statement is:

“The author(s) declared that financial support was received for this work and/or its publication. This work was supported by the Slovenian Research and Innovation Agency (ARIS), research programme P3-0308.”

The original version of this article has been updated.

